# Socio-Demographic Factors and Deformity Among Patients With Leprosy in the Post-elimination Era: A Prospective Study

**DOI:** 10.7759/cureus.114197

**Published:** 2026-08-09

**Authors:** Pritam Kumar, Pratima Verma, Tina Rai, Sourabh Jain, Deepshikha Verma

**Affiliations:** 1 Pathology, Atal Bihari Vajpayee Government Medical College, Vidisha, IND; 2 Microbiology, Chhindwara Institute of Medical Sciences, Chhindwara, IND; 3 Dermatology, Holistic Skin Care, Vidisha, IND; 4 Dermatology, Atal Bihari Vajpayee Government Medical College, Vidisha, IND; 5 Pathology, Gandhi Medical College and Hamidia Hospital, Bhopal, IND

**Keywords:** deformities, early diagnosis, lack of awareness, leprosy, socio-demographic

## Abstract

Introduction: Leprosy, an ancient disease described in the Sushruta Samhita, remains a public health concern in India, despite its elimination at the national level. It is caused by *Mycobacterium leprae *and primarily affects the skin and peripheral nerves and may lead to disability and stigma if untreated. Early detection and timely multidrug therapy are essential for preventing complications and reducing its transmission.

Methods: A prospective observational study was conducted in the Departments of Pathology and Dermatology at Atal Bihari Vajpayee Government Medical College in Vidisha, Madhya Pradesh, India, from April 2024 to July 2025. Thirty suspected cases of leprosy with deformities were evaluated clinically and investigated by slit skin smear and skin biopsy. Cases were categorized according to the World Health Organization (WHO) (National Leprosy Eradication Programme (NLEP)) criteria after obtaining ethical approval and informed consent.

Results: A total of 30 leprosy cases were evaluated, with a maximum of patients aged 15-45 years (18, 60%) and male predominance (21, 70%). The majority of patients were from rural areas, illiterate, and of lower socioeconomic status. The maximum number of patients presented with lesions lasting for more than six months. All cases showed sensory loss with deformity, and 29 (96.6%) had acid-fast bacilli (AFB)-positive slit skin smear. Lepromatous leprosy was the predominant clinical type (20, 66.6%), confirmed by histopathology and classified by the Ridley-Jopling classification.

Conclusions: Leprosy remains a concern despite its elimination status due to continued new cases and deformities. Early diagnosis, timely treatment, awareness, and proper follow-up are essential to prevent disability and reduce transmission.

## Introduction

Leprosy is an ancient disease in India, and its earliest description is found in the Sushruta Samhita, a classical Ayurvedic text written around 600 BC [1]. It is caused by *Mycobacterium leprae*, an acid-fast bacillus (AFB). Although leprosy has been eliminated as a public health problem at the national level in India, it remains a significant public health concern. The disease primarily affects the skin and peripheral nerves, leading to disability, deformity, psychological distress, and social stigma when diagnosis and treatment are delayed. Early case detection, prompt initiation of multidrug therapy, regular follow-up, and self-care are essential for preventing permanent complications and reducing disease transmission. The continued occurrence of new cases in India, which contributes a substantial proportion of the global leprosy burden, highlights the need to identify the socio-demographic factors associated with leprosy. Such evidence is essential for strengthening prevention, promoting early diagnosis, and improving disease control strategies [2,3].

Aim

This study aimed to describe the socio-demographic profile, clinical spectrum, and pattern of deformities among newly diagnosed leprosy patients presenting with deformities at a tertiary care center.

## Materials and methods

This prospective observational study was conducted in the Department of Pathology in collaboration with the Department of Dermatology at Atal Bihari Vajpayee Government Medical College in Vidisha, Madhya Pradesh, India, during the study period from April 2024 to July 2025 (duration: 16 months). Informed consent was obtained from the patients, and ethical permission was obtained from the Institutional Ethical Committee of Atal Bihari Vajpayee Government Medical College (approval number: 04-41/IEC/ABVGMC/Vidisha/2024) before the commencement of the study.

Samples were collected from patients who presented at the Outpatient Department/Inpatient Department (OPD/IPD) of Dermatology and showed features of deformity after clinical examination, and their socio-demographic history and disease-related history were taken. This was followed by slit skin smear examination done from four sites. Bacillary index was calculated according to the bacillary index logarithm scale [4], and detection of AFB by microscopy and clinical diagnosis was made according to the World Health Organization (WHO) (National Leprosy Eradication Programme (NLEP)) classification of leprosy [5]. The findings were recorded and analyzed, and skin punch biopsies were taken from a representative area for histopathology confirmation and subtyping according to the Ridley-Jopling classification [6]. All parameters of socio-demographic factors and clinical examination were recorded and analyzed. Confounding factors that may influence the occurrence of deformities, including age, disease duration, leprosy type, treatment delay, nerve involvement, lepra reactions, treatment adherence, socioeconomic status, education, occupation, and healthcare-seeking behaviour, were considered during the study. Clinical assessment was performed at the time of presentation, and deformities were recorded only if they were present at baseline before treatment initiation. The study did not include longitudinal follow-up to assess the occurrence of new deformities after enrollment.

Inclusion criteria

All newly diagnosed, previously untreated patients with clinically suspected leprosy who presented with one or more leprosy-related deformities at the time of their initial evaluation in the Dermatology OPD/IPD and who provided written informed consent were included in the study.

Exclusion criteria

Patients who refused to provide informed consent, patients with deformities attributable to causes other than leprosy (such as congenital anomalies, trauma, diabetes mellitus, stroke, or other neurological disorders), patients already receiving multidrug therapy, and patients who developed deformities after enrolment during the study period were excluded from the analysis.

Sample size

All consecutive patients meeting the inclusion criteria during the study period were included. A total of 30 cases were available and included in the study.

Statistical analysis

The collected data was entered and analyzed using Microsoft Excel (2011 version) (Microsoft Corp., Redmond, WA, USA). Socio-demographic variables were expressed as frequency and percentage. The distribution of socio-demographic characteristics, clinical features, slit skin smear findings, histopathological findings, and patterns of deformities among newly diagnosed cases of leprosy patients were analyzed and presented in tables. Due to the limited sample size, only descriptive analysis was performed, and no inferential statistical tests were applied.

## Results

In the present study, 30 new cases of leprosy were clinically diagnosed and confirmed on histopathology. Table 1 shows the various socio-demographic parameters, including age distribution (in years), with the maximum number of patients, i.e., 18 (60%), in the 15-45-year age group and the minimum (5, 16.6%) in the age group of 61-90 years. The youngest patient observed was 17 years old, while the oldest patient was 74 years old. Gender-wise, there were 21 males (70%) and nine females (30%) constituting a 7:3 male-to-female ratio in the study population. Among the study participants, 17 (56.6%) were from rural areas, whereas 13 (43.3%) were from urban areas. The maximum number of patients in the study were illiterate (16, 53.3%), and 14 (46.6%) came from lower socioeconomic status. As regards their occupation, farmers (10, 33.3%) comprised the majority of patients, followed by laborers (9, 30%), businessmen/women (4, 13.3%), housewives (2, 6.7%), and others (5, 16.6%). Taking the duration of disease into consideration, 25 (83.3%) patients have skin lesions and other related complaints for more than six months. On analyzing the presenting complaints of patients, all of them have skin lesions with numbness and hypoesthesia with deformity, of whom 23 (76.6%) cases have more than five lesions and seven (23.3%) cases have less than five lesions. Slit skin smear examination was done from four sites, both ear lobes and bilateral lateral one-third of the eyebrow, which showed AFB positivity (multibacillary) in 29 (96.6%) cases and AFB negativity (paucibacillary) in one (3.3%) case.

**Table 1:** Various socio-demographic parameters of new cases of leprosy patients with deformity. AFB: acid-fast bacilli; MB: multibacillary; PB: paucibacillary

Parameters	No. of patients	Percentage
Age in years		
0-14	0	0
15-45	18	60
46-60	7	23.3
61-90	5	16.6
Gender		
Male	21	70
Female	9	30
Place of residence		
Rural	17	56.6
Urban	13	43.3
Education status		
Illiterate	16	53.3
Literate	14	46.6
Socioeconomic status		
Lower class	14	46.6
Middle class	9	30
Upper class	7	23.3
Occupation		
Farmers	10	33.3
Laborers	9	30
Businessmen/women	4	13.3
Housewives	2	6.7
Others	5	16.6
Duration of disease		
Less than 6 months	5	16.6
More than 6 months	25	83.3
No. of lesion		
1-5	7	23.3
>5	23	76.6
AFB slit skin smear		
Positive (MB)	29	96.6
Negative (PB)	1	3.3

**Figure 1:**
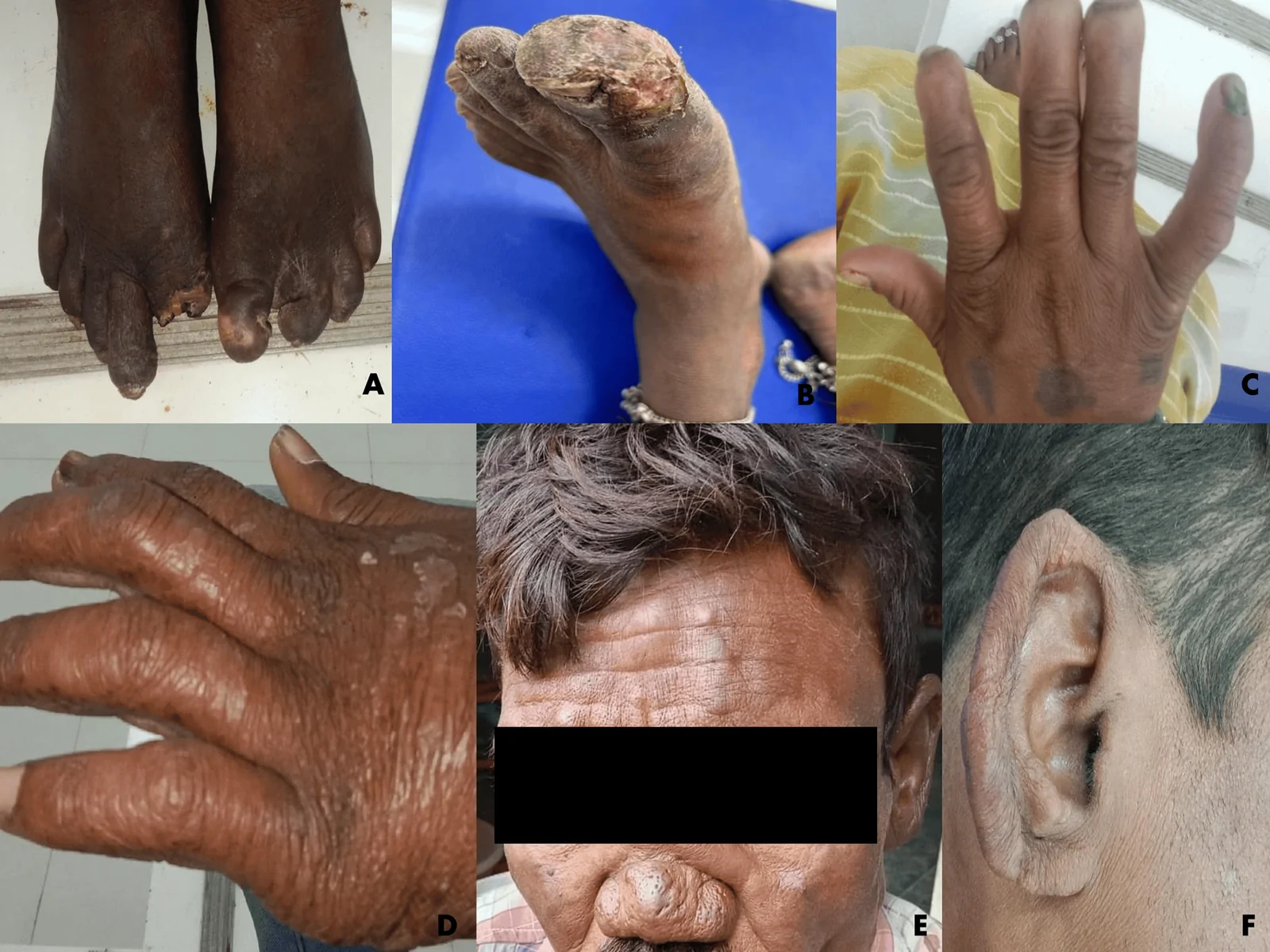
(A-F) Spectrum of various deformities in leprosy patients: (A) absorption of digits, (B) ulcer of the foot, (C) banana fingers, (D) claw hand, (E) leonine facies, and (F) Buddha ear.

In the present study, deformity among clinically diagnosed cases of leprosy (Table 2) showed distribution predominantly as ulcers over the hand and foot in seven (23.3%) cases, followed by absorption of digits in six (20%), banana finger in four (13.3%), nerve thickening in three (10%), claw hand in three (10%), madarosis in two (6.7%), Buddha ear in two (6.7%), leonine facies in two (6.7%), and foot drop in one (3.3%), respectively. Figure 1A-1F shows the various types of clinical presentation of deformity in patients.

**Table 2:** Spectrum of the various types of deformities in leprosy cases.

Deformity	No. of cases	Percentage
Madarosis	2	6.7
Buddha ear	2	6.7
Claw hand	3	10
Foot drop	1	3.3
Leonine facies	2	6.7
Absorption of digits	6	20
Nerve thickening	3	10
Banana finger	4	13.3
Ulcer	7	23.3
Total	30	100

The present study shows the spectrum of clinically diagnosed cases on the basis of WHO (NLEP) classification as follows: the maximum number of cases belonged to lepromatous leprosy (20, 66.6%), followed by borderline lepromatous leprosy (6, 20%), borderline-borderline leprosy (2, 6.7%), and borderline tuberculoid leprosy (2, 6.7%), respectively (Table 3).

**Table 3:** Spectrum of clinical diagnosis of the study participants on the basis of WHO (NLEP) classification. WHO: World Health Organization; NLEP: National Leprosy Eradication Programme

Clinical diagnosis	No. of cases	Percentage
Borderline tuberculoid leprosy	2	6.7
Borderline-borderline leprosy	2	6.7
Borderline lepromatous leprosy	6	20
Lepromatous leprosy	20	66.6
Total	30	100

## Discussion

Leprosy, also known as Hansen's disease, was referred to as Kushta Roga in ancient India. The term Kushta originates from the Sanskrit word Kushnati, meaning "to destroy or eat away," reflecting the destructive nature of the disease [7]. Deformity refers to any abnormality or loss of normal anatomical structure or physiological function. In leprosy, deformities may result from visible or hidden impairments. Primary impairments occur directly due to the disease, such as madarosis or Buddha ear, whereas secondary impairments develop as complications, such as ulcers and absorption of digits.

This study aimed to assess the pattern of deformities among newly diagnosed cases of leprosy patients in the post-elimination era. Evaluating disability status is important for improving disease control strategies, reducing deformities, and addressing the stigma associated with leprosy. Despite progress through the NLEP, early case detection, and multidrug therapy, leprosy still continues to remain a public health concern in India, highlighting the need for ongoing surveillance and effective management [8].

Leprosy being a chronic disease is one of the major public health concerns due to the social stigma and the associated disability. In this study, the age, gender, place of residence, educational status, socioeconomic status, and occupation of leprosy patients with deformity were reported and analyzed.

In the present study, patients' age ranged from 17 to 74 years with a maximum, 60%, in the 15-45-year age group. The age distribution of the present study was comparable with other studies like Mahajan et al. [9] and Patel et al. [10]; this could be due to more chances of exposure, opportunities for infection, and increased awareness regarding seeking medical advice in this age group. Regarding the gender-wise distribution, the male-to-female ratio was 7:3, and a similar pattern of male predominance was noted in the study on a par with Mangala et al. [11] who reported 63.71% males and 36.28% females and Sanker et al. [12] who found 69% males and 31% females. Males often have more chances to acquire infection because they have greater mobility and are more in contact with large groups of people. Females may be affected less due to socioeconomic and cultural barriers which leads to lower reporting in healthcare access.

In the present study, among the study participants, 56.6% are from rural areas and 43.3% from urban areas. Our findings are consistent with those of Doshi et al. [13], who reported that 73.3% of participants were from rural areas. In contrast, Govindarajulu et al. [14] found that 64% of participants were from urban areas.

In the present study, the majority of patients were illiterate (53.3%) in education, were farmers (33.3%) and laborers (30%) by occupation, and belonged to lower socioeconomic status (46.6%). The results were comparable with other studies like Verma et al. [15], Doshi et al. [13], and Mehta et al. [16].

Leprosy and related deformities were more common among laborers and farmers, which highlight the need for targeted awareness programs and early detection strategies. Lower educational and socioeconomic status among patients may contribute to poor disease knowledge, delayed healthcare seeking, and increased transmission due to unfavorable living conditions. In the present study, the majority of patients (83.3%) had an illness duration of more than six months to one year, similar to results reported by Krishna et al. [17] at 65.8%. In the present study, 76.6% of patients presented with more than five skin lesions. This finding is consistent with the high proportion of multibacillary cases, as 96.6% of the patients were AFB-positive. The greater number of skin lesions is indicative of a higher bacillary load and is commonly associated with multibacillary leprosy. Comparable findings were reported by Mahajan et al. [9] (54.01%) and Mehta et al. [16] (88.9%). The increased number of multibacillary cases may be due to delayed diagnosis, emphasizing the need for early case detection and stronger leprosy control measures. In the present study, taking into account the clinical diagnosis spectrum based on the number of lesion and AFB smear status, majority of cases were lepromatous leprosy (66.6%), followed by borderline lepromatous leprosy at 20%, borderline-borderline leprosy at 6.7%, and borderline tuberculoid leprosy at 6.7%. Several studies by Thakkar and Patel [18] observed that the maximum cases occur in the borderline group which indicates the unstable immune status of borderline leprosy.

Deformities in leprosy primarily result from damage to the peripheral nerves caused by *Mycobacterium leprae*. The involvement of sensory, motor, and autonomic nerve fibers leads to loss of sensation, muscle weakness, and autonomic dysfunction. Direct infiltration of the skin and mucous membranes by the *Mycobacterium leprae* bacilli contributes to the development of deformities and associated clinical manifestations. In tuberculoid leprosy, early inflammatory nerve damage causes compression and loss of function, worsened by ulcers and infections. Lepromatous leprosy causes gradual damage to sensory and autonomic nerves, leading to deformities. Borderline leprosy has extensive nerve involvement and a higher risk of severe disability.

In the present study, madarosis (loss of eyebrows) was observed in 6.7% of the patients. This finding is likely related to the higher proportion of patients with lepromatous leprosy. A similar study by Raghavendra et al. [19] reported that only 2% of leprosy patients had madarosis deformity. In the present study, 10% of patients presented with claw hand deformity. A similar study by Mangala et al. [11] reported that 32.7% had claw hand deformity. Hand deformities in leprosy result from damage to motor nerve fibers supplying the hand muscles. The ulnar nerve is most frequently affected, followed by the median nerve, causing ulnar claw hand and total claw hand deformities. In the present study, foot drop was observed in 3.3% of the patients. A study by Schipper et al. [20] reported that 2% of leprosy patients develop foot drop because of damage to the common peroneal nerve in the popliteal region. The low occurrence of cases of foot drop in our study could be a normal variation in the clinical presentation. In the present study, 23.3% of patients presented with ulcers over the foot and hands. A study done by Jain et al. [21] reported 34.6% of ulcers of the foot as deformity in leprosy patients. The reason for this increased incidence of ulcers in this study was due to the fact that a large proportion of cases had anesthesia of the feet and foot ulcers which was the most common foot problem seen in leprosy patients. In the present study, 10% of patients presented with nerve involvement; this proportion was lower compared to the study by Kadam et al. [22], which reported a prevalence of 50%. In the present study, two patients had leonine facies (6.7%), two patients had Buddha ears (6.7%), six patients had amputation of digits (20%), and four patients had banana fingers (13.3%), respectively.

Different patterns of deformities indicate delayed diagnosis and highlight the need for greater community awareness to promote the early detection and timely treatment of leprosy, thereby preventing disability and reducing disease transmission. Contributing factors include delayed case detection, limited healthcare resources, poor adherence to multidrug therapy, inadequate health education, delayed healthcare-seeking behavior, and reliance on traditional healers. The burden is often higher among people from lower socioeconomic backgrounds due to poor awareness and limited access to healthcare services. Strengthening surveillance, improving treatment compliance, and conducting targeted awareness campaigns in high-risk communities are essential to reduce stigma, encourage early treatment, and prevent deformities and disability [23].

Limitations

The present study has limitations due to its small sample size and single-center hospital-based design, which may restrict the applicability of findings. Only newly diagnosed leprosy patients with deformities were included, limiting comparison with patients without deformities. Lack of long-term follow-up and limited statistical analysis restricted the evaluation of treatment outcomes and independent risk factors. Further multicenter studies with longer follow-up are needed to better understand the factors associated with leprosy-related deformities.

## Conclusions

Despite achieving elimination as a public health problem, leprosy remains an important health concern due to the continued occurrence of new cases, particularly among vulnerable populations. The predominance of multibacillary cases and the presence of deformities emphasize the need for early diagnosis, timely treatment, and regular follow-up. Strengthening community awareness, promoting self-care practices, and ensuring accurate clinical and histopathological evaluation are essential to reduce disease transmission, prevent disability, and improve patient outcomes.
